# VopE, a *Vibrio cholerae* Type III Effector, Attenuates the Activation of CWI-MAPK Pathway in Yeast Model System

**DOI:** 10.3389/fcimb.2017.00082

**Published:** 2017-03-20

**Authors:** Leela K. Bankapalli, Rahul C. Mishra, Saumya Raychaudhuri

**Affiliations:** Molecular Biology and Microbial Physiology, Institute of Microbial TechnologyChandigarh, India

**Keywords:** *Vibrio cholerae*, T3SS effector, GAPs, suppression analysis, yeast mitochondria, stressors, MAPK signaling pathway

## Abstract

VopE, a mitochondrial targeting T3SS effector protein of *Vibrio cholerae*, perturbs innate immunity by modulating mitochondrial dynamics. In the current study, ectopic expression of VopE was found to be toxic in a yeast model system and toxicity was further aggravated in the presence of various stressors. Interestingly, a VopE variant lacking predicted mitochondrial targeting sequence (MTS) also exhibited partial lethality in the yeast system. With the aid of yeast genetic tools and different stressors, we have demonstrated that VopE and its derivative VopE^ΔMTS^ modulate cell wall integrity (CWI-MAPK) signaling pathway and have identified several critical residues contributing to the lethality of VopE. Furthermore, co-expression of two effectors VopE^ΔMTS^ and VopX, interfering with the CWI-MAPK cellular pathway can partially suppress the VopX mediated yeast growth inhibition. Taken together, these results suggest that VopE alters signaling through the CWI-MAPK pathway, and demonstrates the usefulness of yeast model system to gain additional insights on the functionality of VopE.

## Introduction

The effectiveness of a microbe as a pathogen is largely dependent on its ability to subvert the host defense mechanism. In many bacterial pathogens, the type 3 secretion system (T3SS) and associated effector proteins are utilized to hijack host immune surveillance, further allowing survival of the bacteria in hostile host environments. Multiple lines of converging evidences clearly demonstrate the evolution of unique functional domains and motifs in several T3SS effector proteins, that empower diverse pathogens to usurp disparate host cellular processes (Dean, 2011; Salomon et al., 2013).

A T3SS secretion system and 11 bona fide effector proteins with unique structural and functional features have been reported in *Vibrio cholerae* strains belonging to diverse serogroups and role of these effector proteins in pathogenesis have been studied *in vitro* and also *in vivo* using animal and yeast model system (Dziejman et al., 2005; Tam et al., 2007; Tripathi et al., 2010; Alam et al., 2011). One such effector protein VopE, a close homolog of the *Yersinia* T3SS effector protein YopE, disrupts the integrity of tight junctions by modulating actin homeostasis, thus promoting pathogenecity of *V. cholerae* strain AM-19226 (Tam et al., 2010; Shin et al., 2011). Furthermore, it has been demonstrated that VopE by virtue of a typical mitochondrial targeting sequence (MTS) at the N-terminus localizes to the mitochondria where it alters mitochondrial dynamics and modulates innate immune signaling by interfering with Miro GTPases (Suzuki et al., 2014). Sequence analysis reveals the existence of GTPase-activating protein (GAP) domain at the C-terminus of VopE (Suzuki et al., 2014). Such GAP domain is also reported in other T3SS effector proteins such as YopE (*Yersinia pestis*), SptP (*Salmonella typhimurium*), and ExoS (*Pseudomonas aeruginosa*) which are known to target the actin cytoskeleton in mammalian cells (Busch and Aktories, 2000). Though sequence based structural features predict that VopE shares structural homology with YopE, but VopE does not target actin cytoskeleton (Suzuki et al., 2014).

There is a growing appreciation of the utility of *Saccharomyces cerevisiae*, the budding yeast as a non-mammalian model system to identify and evaluate the functionality of diverse arrays of virulence factors from numerous pathogenic bacteria (Valdivia, 2004; Siggers and Lesser, 2008; Curak et al., 2009; Popa et al., 2016). Simple ectopic expression of virulence factors can lead to a variety of discernable phenotypes in yeast that help to build testable hypotheses regarding their function and roles in pathogenesis. There are seminal evidences where the function of numerous effector proteins (e.g., YopE, SipA, Tarp, VopX etc.) was first evaluated in the yeast model and documented later in mammalian systems (Von Pawel-Rammingen et al., 2000; Lesser and Miller, 2001; Galkin et al., 2002; Kumar et al., 2006; Sisko et al., 2006; Siggers and Lesser, 2008; Alam et al., 2011). There are instances where expression of certain effector proteins gave rise to different phenotypes in yeast system than mammalian system, thereby entailing additional function associated with a particular effector (Siggers and Lesser, 2008). For example expression of Map (T3SS effector protein of *Escherichia coli*) results in the formation of filopodia in mammalian cells but its expression in yeast model promotes the formation of large unbudded cells (Rodríguez-Escudero et al., 2005). Additionally, yeast has also been used as a model to gain new insights on mammalian proteins (Pereira et al., 2012).

The mitogen-activated protein kinase (MAPK) signaling pathways have been studied in great detail in *S. cerevisiae* (Hunter and Plowman, 1997; Gustin et al., 1998). The MAPK signaling pathway has been shown to be targeted by several T3SS effectors of diverse pathogenic bacteria. For example, HOG MAPK pathway of *S. cerevisiae* is targeted by VopA (*Vibrio parahemolyticus*), YopJ (*Yersinia* sp.), and HopX1 (*Pseudomonas syringae*) (Yoon et al., 2003; Trosky et al., 2004; Salomon et al., 2012). YopJ also targets the pheromone response MAPK pathway in the yeast model system (Yoon et al., 2003). The T3SS effector protein of *V. cholerae*, VopX has been reported to interfere with the functioning of the CWI-MAPK pathway (Alam et al., 2011; Seward et al., 2015). In sum, yeast model system reveals that MAPK signaling pathways are potential targets of T3SS effector proteins.

In this study, another T3SS effector protein of *V. cholerae*, VopE has been investigated using yeast as a model system. The results obtained clearly revealed that level of toxicity of VopE is dependent on yeast genetic background and is exacerbated in the presence of various stressors. Though mitochondrial localization of VopE is important for full toxicity, deletion of mitochondrial target sequence (MTS) still retained partial lethality. By employing different stressors coupled with β-galactosidase assay, VopE and its variant (VopE^ΔMTS^) were found to interfere with MAPK pathway primarily affecting the functioning of cell wall integrity (CWI) pathway. Interestingly, screening yeast deletion library related to CWI cellular pathway identified hypersensitive strains. Furthermore, the present work also identified several critical residues contributing to VopE function and unveiled how co-expression of two effectors, VopE^ΔMTS^ and VopX, targeting CWI-MAPK cellular pathway in an opposing manner can suppress the VopX mediated yeast growth inhibition.

## Materials and methods

### Strains and plasmids

The strains and plasmids used in this study are listed in Table 1. *E. coli* strains Nova blue or DH5α were used for general cloning. The *E. coli* strains were propagated at 37°C in Luria Broth (LB) with agitation or on LB agar plates. When appropriate, the growth medium was supplemented with ampicillin (100 μg/ml). *S. cerevisiae* strains were grown at 30°C in YPD [1% (w/v) yeast extract, 2% (w/v) peptone, 2% (w/v) glucose] broth or agar (2%). Media ingredients were purchased from Himedia and Difco. Identity of all BY4741 deletion strains were verified by UP TAG (barcode) confirmation.

**Table 1:** **Strains and plasmids used in this study**.

**Strains/plasmids**	**Genotype/Description**	**Source/References**
*E. coli*		
NovaBlue	*E. coli* K-12, *recA endA, lacI^*q*^, lacY*	Novagen
DH5α	*F– Φ80*lac*ZΔM15 Δ(*lac*ZYA-*arg*F) U169* rec*A1* end*A1* hsd*R17 (rK–, mK+)* pho*A* sup*E44 λ–* thi*-1* gyr*A96* rel*A1*	Microbial Type Culture Collection and Gene Bank (MTCC)
*V. choerae*		
SC110	Non-O1/non-O139 strain of *V. cholerae* serogroup O34	Tripathi et al., 2010
***S. cerevisiae*** **strains**		
BY4741	*MATa; his3Δ 1; leu2Δ 0; met15Δ 0; ura3Δ 0*	Bankapalli et al., 2015
BY4741 Δ*bck1*	*MATa; his3Δ 1; leu2Δ 0; met15Δ 0; ura3Δ 0; bck1Δ::kanMX4*	EUROSCARF
BY4741 Δ*slt2*	*MATa; his3Δ 1; leu2Δ 0; met15Δ 0; ura3Δ 0; slt2Δ::kanMX4*	EUROSCARF
BY4741 Δ*ire1*	*MATa; his3Δ 1; leu2Δ 0; met15Δ 0; ura3Δ 0; ire1Δ::kanMX4*	EUROSCARF
BY4741 Δ*fus3*	*MATa; his3Δ 1; leu2Δ 0; met15Δ 0; ura3Δ 0; fus3Δ::kanMX4*	EUROSCARF
BY4741 Δ*kss1*	*MATa; his3Δ 1; leu2Δ 0; met15Δ 0; ura3Δ 0; kss1Δ::kanMX4*	EUROSCARF
BY4741 Δ*smk1*	*MATa; his3Δ 1; leu2Δ 0; met15Δ 0; ura3Δ 0; smk1Δ::kanMX4*	EUROSCARF
BY4741 Δ*rlm1*	*MATa; his3Δ 1; leu2Δ 0; met15Δ 0; ura3Δ 0; rlm1Δ::kanMX4*	EUROSCARF
BY4741 Δ*kdx1*	*MATa; his3Δ 1; leu2Δ 0; met15Δ 0; ura3Δ 0; kdx1Δ::kanMX4*	EUROSCARF
BY4741 Δ*yca1*	*MATa; his3Δ 1; leu2Δ 0; met15Δ 0; ura3Δ 0; yca1Δ::kanMX4*	EUROSCARF
BY4741 Δ*gem1*	*MATa; his3Δ 1; leu2Δ 0; met15Δ 0; ura3Δ 0; gem1Δ::kanMX4*	EUROSCARF
BY4741 Δ*dnm1*	*MATa; his3Δ 1; leu2Δ 0; met15Δ 0; ura3Δ 0; dnm1Δ::kanMX4*	EUROSCARF
BY4741 Δ*fzo1*	*MATa; his3Δ 1; leu2Δ 0; met15Δ 0; ura3Δ 0; fzo1Δ::kanMX4*	EUROSCARF
BY4741 Δ*mtg1*	*MATa; his3Δ 1; leu2Δ 0; met15Δ 0; ura3Δ 0; mtg1Δ::kanMX4*	EUROSCARF
BY4741 Δ*mtg2*	*MATa; his3Δ 1; leu2Δ 0; met15Δ 0; ura3Δ 0; mtg2Δ::kanMX4*	EUROSCARF
BY4741 Δ*hog1*	*MATa; his3Δ 1; leu2Δ 0; met15Δ 0; ura3Δ 0; hog1Δ::kanMX4*	EUROSCARF
BY4741 Δ*mkk1*	*MATa; his3Δ 1; leu2Δ 0; met15Δ 0; ura3Δ 0; mkk1Δ::kanMX4*	EUROSCARF
BY4741 Δ*mkk2*	*MATa; his3Δ 1; leu2Δ 0; met15Δ 0; ura3Δ 0; mkk2Δ::kanMX4*	EUROSCARF
BY4741 Δ*mgm1*	*MATa; his3Δ 1; leu2Δ 0; met15Δ 0; ura3Δ 0; mgm1Δ::kanMX4*	EUROSCARF
W303-1A	*MATa leu2-3,112 his3-11,15 ade2-1 ura3-1 trp1-1 can1-100 GAL SUC2*	Tripathi et al., 2010
**Plasmids**		
pESCLEU	2μ,*GAL1/GAL10,LEU2*	Agilent Technologies
pGML10	*CEN6/ARSH4,GAL1,LEU2*	RIKEN
pAMU10	*CEN6/ARSH4,ADH1,URA3*	RIKEN
pRS413	*CEN6/ARSH4,TEF,HIS3*	Gift from Dr. Deepak Sharma, Institute of Microbial Technology
pESCLEU-VopE	615 bp *vopE* gene fragment amplified from genomic DNA of *V. cholerae* SC110 with *XhoI/NheI* primers by PCR and cloned into similarly digested pESCLEU under *GAL1* promoter	This study
pESCLEU-VopE^R125A^	VopE harboring A in place of R at position 125	This study
pESCLEU-VopE^Δ24−80^	VopE harboring deletion for 24–80	This study
pESCLEU-VopE^Δ1−80^	VopE harboring deletion for 1–80	This study
pESCLEU-VopE^ΔMTS^	VopE harboring deletion for 1–23	This study
pESCLEU-VopE^ΔMTS R125A^	VopE harboring deletion for 1–23, A in place of R at position 125	This study
pESCLEU-VopE^Δ119−121^	VopE harboring deletion for 119–121	This study
pESCLEU-VopE^Δ159−167^	VopE harboring deletion for 159–167	This study
pESCLEU-VopE^A120G^	VopE harboring G in place of A at 120	This study
pESCLEU-VopE^T129A^	VopE harboring A in place of T at 129	This study
pESCLEU-VopE^Q161A^	VopE harboring A in place of Q at 161	This study
pESCLEU-VopE^G163A^	VopE harboring A in place of G at 163	This study
pESCLEU-VopE^T164A^	VopE harboring A in place of T at 164	This study
pESCLEU-VopE^F110A^	VopE harboring A in place of F at 110	This study
pESCLEU-VopE^F162A^	VopE harboring A in place of F at 162	This study
pESCLEU-VopE^V173A^	VopE harboring A in place of V at 173	This study
pESCLEU-mCherry	*mCherry* gene fragment was inserted into pESCLEU at *XhoI, NheI* sites under *GAL1* promoter	This study
pESCLEU-VopE-mCherry	*vopE-mCherry* fragment was inserted into pESCLEU at *XhoI, NheI* sites under *GAL1* promoter	This study
pESCLEU-VopE^R125A^-mCherry	*vopE*^R125A^*-mCherry* fragment was inserted into pESCLEU at *XhoI, NheI* sites under *GAL1* promoter	This study
pESCLEU-VopE^ΔMTS^-mCherry	*vopE^Δ*MTS*^-mCherry* fragment was inserted into pESCLEU at *XhoI, NheI* sites under *GAL1* promoter	This study
pESCLEU-Gem1	1.99 kb *Gem1* gene fragment amplified from genomic DNA of BY4741 with *NotI/PacI* primers by PCR and cloned into similarly digested pESCLEU under *GAL10* promoter	This study
pESCLEU-VopE-Gem1	*vopE* (*XhoI/NheI*) and *Gem1* (*NotI/PacI*) fragments were cloned in pESCLEU under *GAL1* and *GAL10* promoters, respectively	This study
pESCLEU-VopX	*vopX* obtained by *NotI/PacI* primers inserted into pESCLEU under *GAL10* promoter	This study
pESCLEU-VopE-VopX	vopE (*XhoI/NheI*) and *vopX* (*NotI/PacI*) fragments were cloned in pESCLEU under *GAL1* and *GAL10* promoters respectively	This study
pESCLEU-VopE^ΔMTS^-VopX	*vopE ^Δ*MTS*^* (*XhoI/NheI*) and *vopX* (*NotI/PacI*) fragments were cloned in pESCLEU under *GAL1* and *GAL10* promoters, respectively	This study
pESCLEU-VopE^ΔMTS R125A^-VopX	*vopE*^ΔMTS R125A^ (*XhoI/NheI*) and *vopX* (*NotI/PacI*) fragments were cloned in pESCLEU under *GAL1* and *GAL10* promoters, respectively	This study
pGML10-VopE	615 bp *vopE* gene fragment amplified from pESCLEU-VopE with *BamHI/EcorI* primers by PCR and cloned into similarly digested pGML10 under *GAL1* promoter	This study
pAMU10-VopE	615 bp *vopE* gene fragment amplified from pESCLEU-VopE with *BamHI/EcorI* primers by PCR and cloned into similarly digested pAMU10 under *ADH1* promoter	This study
pRS413-COX4-GFP	Amplified *COX4-GFP* fragment cloned into *XbaI, BamHI* sites of pRS413 under *TEF* promoter	This study
pKT760	HOG 8x*CRE*-*lacZ, TRP1*	Gift from Dr. Guido Sessa, Tel Aviv University, Israel
p1434	CWI 2xRlm1-*lacZ, URA3*	
pMCZ	UPR, UPRE-*lacZ, URA3*	
Pheromone MAPK pathway Reporter plasmid	Pheromone, *P_*FUS1*_-lacZ, URA3*	

The *vopE* gene was amplified from the genomic DNA of *V. cholerae* strain SC110 using the gene specific primers and used to generate recombinant plasmids (Table 1). The conserved residues or regions of *VopE* were either deleted or substituted with alanine or glycine using Stratagene one step mutagenesis kit. The recombinant plasmid pESCLEU-VopE was used as a template for all mutagenesis reactions. The desired constructs were further transformed into *S. cerevisiae* strains.

For localization studies, all VopE constructs were C-terminally tagged with mCherry (from pmCherry-C1, Clonetech) by an overlapping PCR as described earlier (Tripathi et al., 2010). For mitochondrial visualization, *COX4*-*GFP* fragment was constructed by fusing a fragment encoding first 63 bases of *COX4* to a gene encoding GFP (from pGREG575) by overlapping PCR method. This construct was further inserted into pRS413. This recombinant plasmid pCOX4-GFP was transformed into BY4741 to generate reporter strain BY4741-COX4-GFP. For Gem1 over expression, *Gem1* gene was amplified from genomic DNA of BY4741 with *NotI*F/*PacI*R primers, and further inserted into pESCLEU-VopE to generate dual expression plasmid pESCLEU-VopE-Gem1. For VopX expression, *vopX* gene was amplified from the genomic DNA of *V.cholerae* strain SC110 using the gene specific primers, and subsequently inserted into *NotI/PacI* sites of pESCLEU or pESCLEU-VopE to generate recombinant plasmids. For protein detection, VopE constructs were tagged at C-terminus with 3XFLAG. All recombinant plasmids constructed in this study (Table 1) were confirmed by sequencing and maintained in *E. coli* strains.

### Yeast growth and viability assays

Yeast growth assays were performed as described earlier (Tripathi et al., 2010; Bankapalli et al., 2015). Briefly, overnight cultures of all recombinant strains in selective SC^Raf^ medium were diluted and grown again in same media at 30°C until exponential phase (OD_600_ ~ 0.8–1.0). Effect of the expression of VopE, its derivatives and VopX on the growth of *S. cerevisiae* strains was examined by spotting equal number of cells onto SC and SC^Gal^ plates lacking the corresponding auxotrophic markers to maintain the plasmids. When necessary, liquid growth induction was done for 6 h by adding galactose (2%) to cultures before spotting. Growth was monitored after 60–70 h at 30°C and photographed accordingly. Liquid growth assay was done as described earlier (Bankapalli et al., 2015). For yeast viability plating assay (cfu), recombinant stains carrying VopE or its variants were grown overnight in selective SC^Raf^ media. Cultures were again diluted to OD_600_ 0.1 in fresh media and incubated up to OD_600_ ~1.0 where cultures were resuspended in selective SC^Gal^ media. At different times after induction, aliquots were removed, and serial dilutions were plated on selective SC solid media. Plates were incubated for 2–3 days at 30°C before viable colonies were counted (Salomon et al., 2011).

### Yeast β-galactosidase assay

*S. cerevisiae* strain BY4741 harboring pESCLEU-VopE (BY4741-VopE) was transformed with various *lacZ* reporter plasmids as listed in Table 1 (2XRlm1-*lacZ*, P_FUS1_-*lacZ*, and UPRE-*lacZ*). The HOG pathway related *lacZ* reporter plasmid (HOG 8X*CRE*-*lacZ*) was transformed into W303-1A strain harboring pESCLEU-VopE (W303-VopE). All the plasmids were obtained as gift from Dr. Guido Sessa, Tel Aviv University, Tel Aviv, Israel (Salomon et al., 2012). For β-galactosidase assay, we followed the published protocol (Salomon et al., 2012). Briefly, overnight cultures of recombinant strains were diluted in selective medium containing raffinose (2%) and grown to OD_600_ 0.6–0.9. Galactose (2%) was added and incubated for 6 h. Equal amount of cultures were then distributed into two tubes. In one set, stressors were added and kept for stipulated period as given. To activate the HOG pathway, 0.5 M NaCl for 1 h; to activate the CWI pathway, 3 mM caffeine for 4 h; to stimulate the UPR, 2 mM DTT for 4 h; to induce the pheromone signaling pathway, 5 μM α-factor for 2 h. Cells were finally harvested and subjected to rapid permeabilization procedure as described earlier (Kippert, 1995). Activity is reported as percentage of Miller units.

### Preparation of yeast extracts and immunoblot analysis

The immunoblot analysis was carried out based on previous work (Tripathi et al., 2013; Bankapalli et al., 2015). All the recombinant yeast strains carrying wild type VopE and its congeners were grown in selective SC^Raf^ medium at 30°C until mid-log phase. The cultures were then diluted in selective induction media (SC^Gal^). After 6 h of induction, cultures were pelleted and whole cell protein extracts were prepared by adopting a published protocol (Zhang et al., 2011). Equal amount of protein samples were fractionated by SDS-polyacrylamide gel electrophoresis using 12% polyacrylamide gel and transferred to Immobilon–P Transfer Membrane (Millipore). Membrane was probed with either Monoclonal anti-FLAG M2-Peroxidase (HRP) Clone M2 (Sigma) antibody or anti-Porin (yeast mitochondria) Monoclonal Antibody (Invitrogen, Mitosciences). The primary antibody was detected using a horseradish peroxidase conjugated anti-mouse antibody (Millipore) and blot was developed with Luminata™ Forte Western HRP substrate.

### Yeast confocal microscopy and subcellular fractionation

To investigate the localization of VopE and its mutagenized congeners, recombinant yeast strains harboring these constructs (Table 1) were harvested after galactose induction. Samples were observed by Confocal Laser Scanning Microscope (Nikon A1R). For isolation of mitochondria, yeast cells containing vector (pESCLEU), or VopE were grown in selective SC media and grown again in 500 ml of fresh media till OD_600_ was 1.5. Subsequently, cells were harvested and expression of VopE was induced by re-suspending the pellet in 500 ml of selective SC^Gal^ media for 6 h. Purification of yeast mitochondria was performed according to standard procedure (Gregg et al., 2009).

## Results

### Ectopic expression of VopE in *S. cerevisiae* affects cell growth

**Figure 1:**
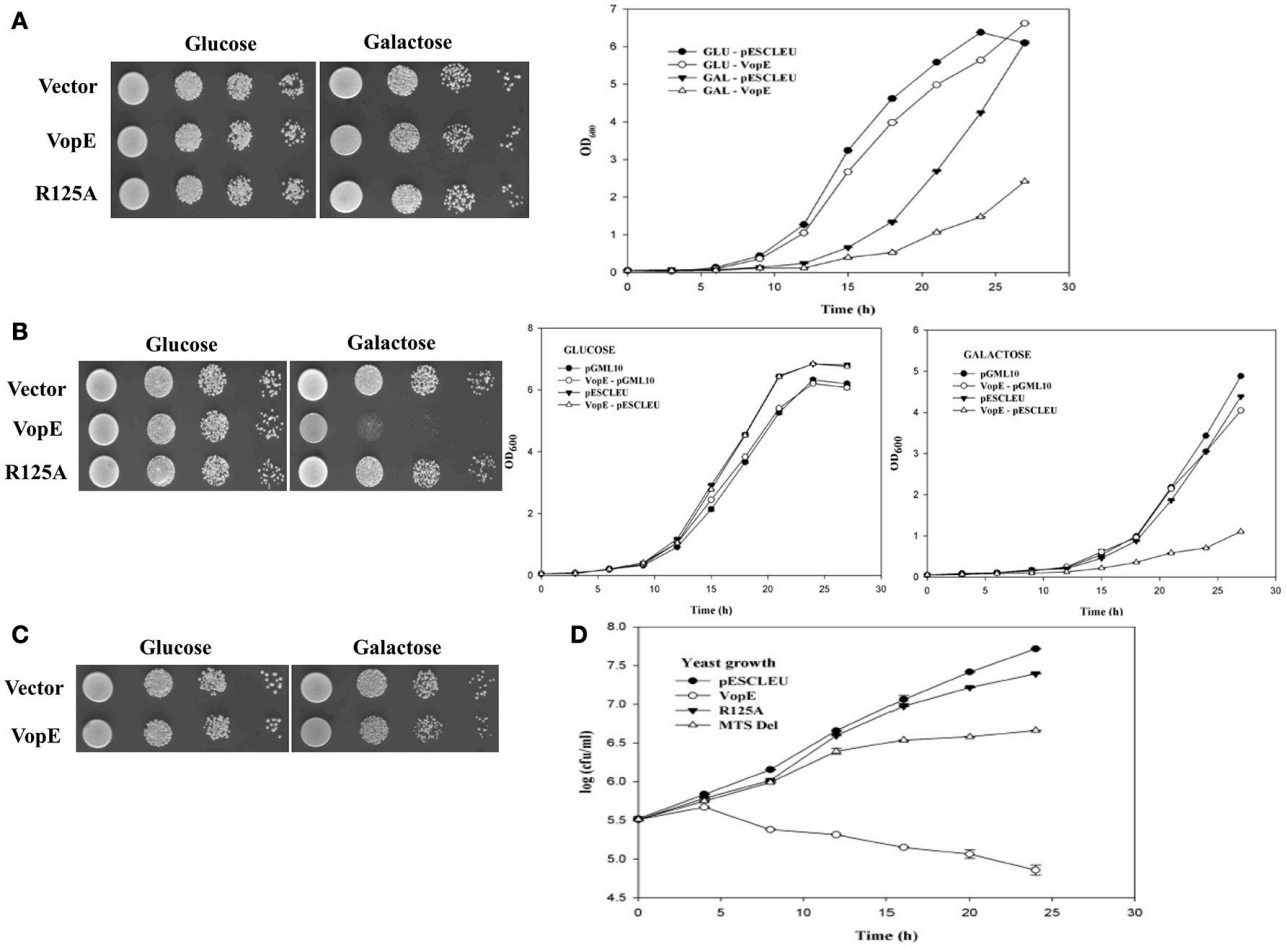
**Effect of VopE on Yeast growth. (A)** W303-1A transformants carrying VopE or VopE^R125A^ under control of *GAL1* promoter were subjected to spotting on selective solid media containing glucose (repressor) or galactose (inducer). Liquid growth assay shown on right panel. Yeast harboring pESCLEU (high copy vector) treated as control. **(B)** Similar experiment was performed in BY4741 genetic background with pESCLEU cloning vector. **(C)** BY4741 transformants harboring pGML10 (low copy vector) or VopE were subjected to spotting under selective solid media containing glucose or galactose conditions. Liquid growth assay shown in **(B)** right panel. **(D)** For yeast growth viability analysis, BY4741 strains carrying VopE constructs were sampled at each time point and 10-fold serial dilutions were plated on glucose plate to count viable cells. Error bar indicates standard deviation from mean. Each experiment repeated three times with similar results.

Several *V. cholerae* strains belonging to non-O1, non-O139 serogroups (laboratory collection) were examined for the presence of the *vopE gene*. The gene fragment was PCR amplified and cloned under the control of *GAL1* promoter in the high copy number vector pESCLEU to generate recombinant plasmid pESCLEU-VopE. Next, VopE (A33_1662) was ectopically expressed in a *S. cerevisiae* strain W303-1A. Under inducing condition, spotting on solid agar media exhibited no growth inhibition, where as in liquid media strong growth inhibition was observed (Figure 1A, left and right panels). As reported, certain effectors exhibit an altered toxicity in solid and liquid growth assay conditions in yeast model system (Kramer et al., 2007; Siggers and Lesser, 2008; Antic et al., 2014). Interestingly, it has been documented that effector driven growth inhibition is dependent on different yeast genetic backgrounds (Rodríguez-Pachón et al., 2002; Bénit et al., 2010; Salomon et al., 2011). To ascertain the function of VopE in another genetic background, we transformed the pESCLEU-VopE recombinant construct into strain BY4741. An increase in lethality of VopE in BY4741 genetic background over that in W303-1A was observed (Figure 1B left and right panels).

**Figure 2:**
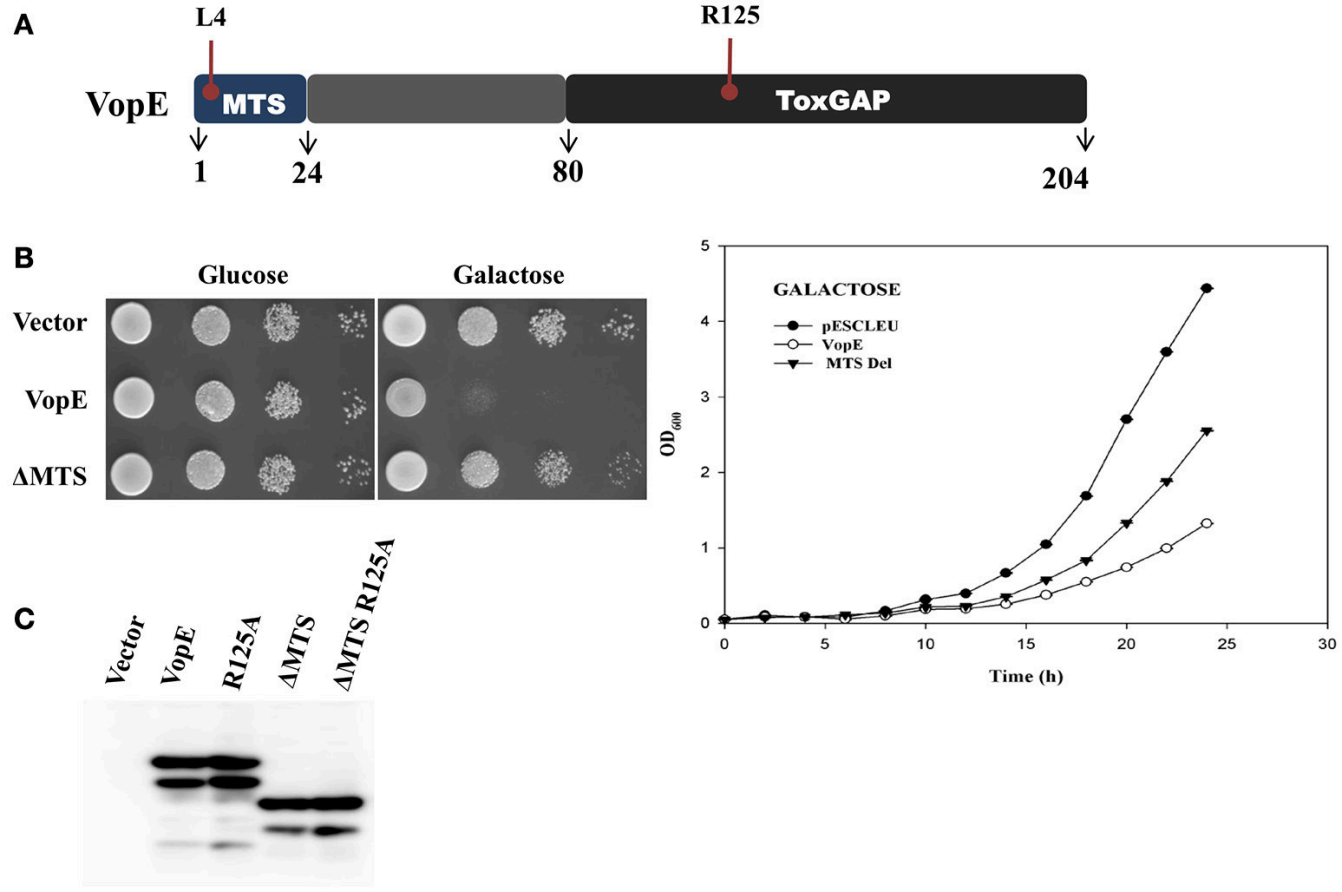
**VopE^**ΔMTS**^ induced yeast growth inhibition. (A)** Schematic representation of VopE domain or motif **(B)** BY4741 strains carrying pESCLEU or VopE^ΔMTS^ were spotted on solid selection media containing glucose or galactose (left panel). Same cultures also inoculated in liquid selection media containing galactose (right panel). **(C)** *In vivo* stability of VopE-3XFLAG and its derivatives confirmed by immunoblot using anti-FLAG antibody.

Activity of bacterial effectors carrying GAP domain largely depend on arginine residue, referred as the arginine finger. VopE is a member of bacterial GAP effector family and also carries a conserved arginine along with additional GAP domain features. To ascertain the contribution of arginine at position 125 in VopE mediated toxicity in yeast system, we exchanged arginine with alanine and evaluated the lethality of recombinant VopE^R125A^ variant. The data clearly demonstrated significant loss in lethality of VopE^R125A^ variant, thereby underscoring the importance of arginine residue in the functionality of VopE in yeast system (Figure 1B left panel). To investigate *in vivo* protein stability, wild type and VopE^R125A^ were C-terminally tagged with FLAG epitope and western blot analysis using anti-FLAG antibody was carried out (Zhang et al., 2011; Tripathi et al., 2013). The results indicated that VopE and VopE^R125A^ were found to be stable (Figure 2C). The viability of yeast carrying VopE or VopE^R125A^ was also determined by CFU assay at different time periods up to 24 h. A gradual decrease in number of viable cells suggesting that growth inhibition caused by VopE is due to loss of viability (Figure 1D).

Although, bacteria encode different T3SS effector proteins, quantity of each effector translocated into host varies in amount during infection. Earlier studies have clearly demonstrated that low level expression of effector proteins increases the specificity while high level expression promotes sensitivity of inhibition in yeast model system (Slagowski et al., 2008). In some cases, it is also surmised that high level expression may result in non-specific toxic effects (Salomon et al., 2012). Therefore, we wanted to ascertain whether low level expression of VopE maintains similar toxicity as presented in the preceding section, the gene encoding wild type VopE was cloned in pGML10, a low copy number vector (Table 1). Unlike yeast harboring pESCLEU-VopE construct, recombinant yeast cells transformed with pGML10-VopE show mild growth defect, further indicating that VopE toxicity depends on the level of expression (Figures 1C and 1B right panel). As evidenced, several effector proteins exhibit toxicity only when expressed at high level (Slagowski et al., 2008). During analyzing the function of various *Legionella pneumophila* effectors in yeast system, it appears that toxicity of some effectors manifests only at high level while others are independent of the level of expression (Havey and Roy, 2015).

### VopE variant lacking mitochondrial target sequence (VopE^ΔMTS^) retains partial lethality

VopE contains predicted MTS spanning N-terminal 23 amino acids and removal of this stretch has been reported to affect VopE localization to mitochondria (Suzuki et al., 2014). To investigate the cellular fate of VopE variant lacking MTS (henceforth known as VopE^ΔMTS^), growth of recombinant strain BY4741-VopE^ΔMTS^ carries a VopE^ΔMTS^ in high copy number vector was examined under inducing condition. Solid agar spotting data indicate that (VopE^ΔMTS^) retains some ability to intoxicate yeast cells albeit less than wild type protein VopE (Figure 2B, left panel). The spotting data were further corroborated by liquid growth assay where a similar trend of growth inhibition was noticed (Figure 2B, right panel). To ensure *in vivo* stability of VopE^ΔMTS^, western blot was performed by using anti-FLAG antibody (Figure 2C). In growth viability assay, number of growing cells increased for first 12 h, and then maintained as constant number of viable cells (Figure 1D).

### Identification of additional conserved structural unit contributing VopE activity

**Figure 3:**
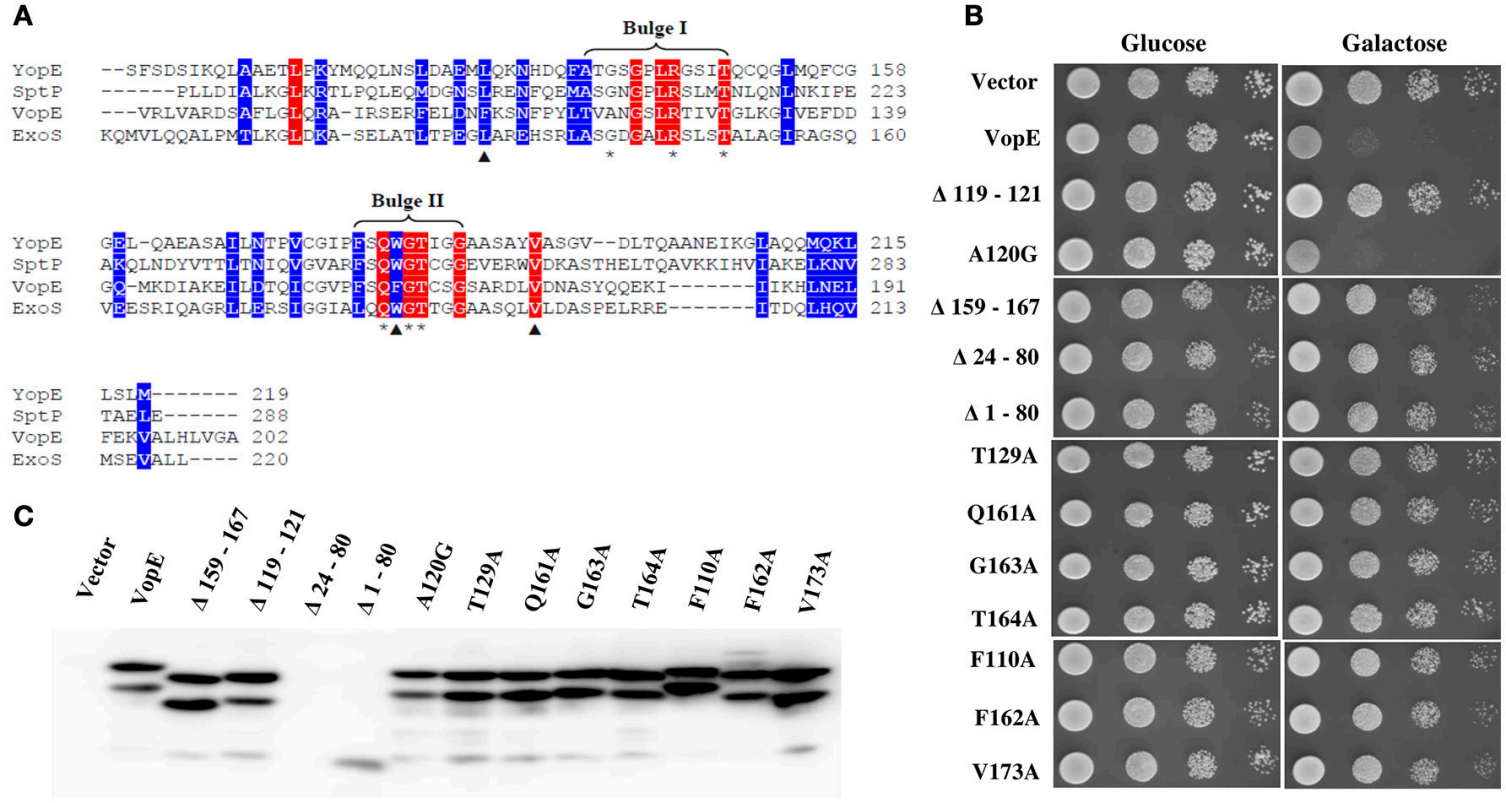
**Identification of residues and regions of VopE contributed in yeast toxicity. (A)** Alignment of VopE along with other bacterial GTPase-activating protein sequences as carried out by Clustal Omega. Conserved and semi-conserved amino acid positions are highlighted with red and blue respectively. Predicted amino acids (^*^) involved in multiple interactions with unknown yeast target protein(s) or selected hydrophobic amino acids (▴) are substituted with alanine or glycine. The GenBank identifiers and species name are as follows: YopE [31795304|*Yersinia pestis*]; SptP[16421425|*Salmonella typhimurium str. LT2*]; VopE [254285224|*Vibrio cholerae* AM19226]; ExoS [450297| *Pseudomonas aeruginosa*]. **(B)** BY4741 cultures carrying a vector, either pESCLEU or VopE variants were 10-folded serial diluted and subjected to spotting on selective solid media containing glucose or galactose. **(C)** Expression of VopE-3XFLAG variants verified by western blot using anti-FLAG antibody.

Structural and sequence analysis reveal certain unique features essential for optimal performance of different bacterial GTPase-activating proteins (GAPs) (Litvak and Selinger, 2003). For example, there are several conserved residues and two bulge regions (I & II) in bacterial GAPs whose mutual interactions maintain the precise orientation of the critical arginine and catalytic efficiency of the GAPs. The catalytic arginine residue within the bulge I interacts with the nucleotide phosphate and with the conserved residues of bulge II (Stebbins and Galan, 2000; Wurtele et al., 2001; Litvak and Selinger, 2003). Sequence alignment of VopE with other bacterial GAPs identified bulge II region (Figure 3A). To examine the importance of this region in VopE function, the bulge II region (amino acids 159–167) was deleted and the activity of VopE^Δ159−167^ variant was examined in yeast strain BY4741. A loss of lethality was observed in the yeast strain carrying the VopE^Δ159−167^ variant (Figure 3B) further corroborating the importance of bulge II region in VopE function. Next, we turned our attention on other amino acids (V^119^, A^120^, N^121^, T^129^, Q^161^, G^163^, T^164^) predicted to involve in network of multiple interactions (Figure 3A). A series of VopE variants carrying alanine substitutions (T129A, Q161A, G163A, and T164A), glycine substitution (A120G) or VAN deletion (Δ119–121) were constructed and their effect was examined in yeast. Significant loss in lethality of VopE alanine (T129A, Q161A, G163A, T164A) and deleted (ΔVAN) variants (Figure 3B), thereby indicating the significance of these interacting residues in maintaining VopE function in addition to arginine^125^ residue. It should be noted that toxicity of VopE variant harboring glycine at position 120 (VopE^A120G^) remains unperturbed.

In addition to the residues posited in bulge I and II regions, there are residues participating in the formation of hydrophobic core in the GAP domain of SptP (Stebbins and Galan, 2000). Based on Clustal Omega alignment of VopE with SptP, ExoS, and YopE, three amino acids such as F^110^, F^162^, and V^173^ were selected that might contribute to the formation of hydrophobic core of VopE (Figure 3A). Subsequent replacement of these amino acids with alanine resulted in loss of functionality of VopE (Figure 3B). Interestingly, non-conserved region might also contribute to the function of any given protein (Sampath et al., 2003). Indeed, when a non-conserved region spanning 24–80 amino acids (Figure 2A) was deleted, the resultant VopE^Δ 24–80^ variant was found to be non-lethal in yeast model system (Figure 3B). Collectively, our effort led to the identification of several GAP domain associated residues and non-conserved region contributing to the functionality of VopE. To examine *in vivo* stability, western blot analysis of recombinant yeast strains harboring wild type and different VopE variants was carried out by using anti-FLAG antibody. We found that all the VopE derivatives were stable under *in vivo* condition except VopE^Δ24–80^ and VopE^Δ 1–80^ (Figure 3C).

### Mitochondrial localization of VopE in *S. cerevisiae*

**Figure 4:**
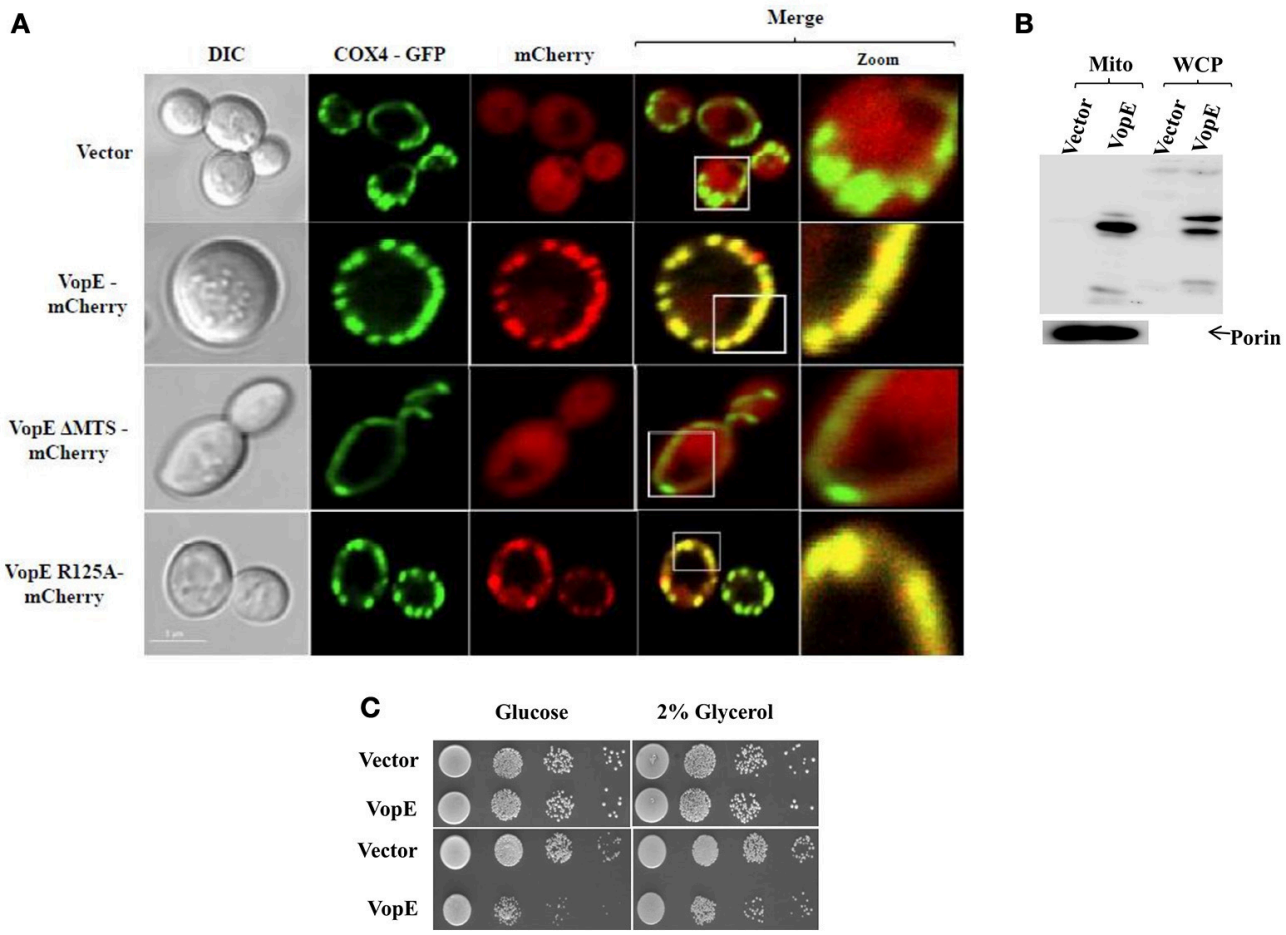
**VopE targeted to yeast mitochondria and does not impair respiration. (A)** Plasmids encoding mCherry or VopE-mCherry variants were introduced into yeast reporter strain BY4741-COX4-GFP. Mid-log phase cells were induced for 6 h by the addition of galactose. Mitochondria were visualized by COX4-GFP (targeting to mitochondrial matrix). Cells were examined by confocal laser scanning microscopy to monitor the localization patterns of VopE-mCherry constructs. Scale bar equals to 5 μm. **(B)** Detection of VopE-3XFLAG in mitochondrial fraction and whole cell lysate of 6 h galactose induced cultures with anti-FLAG antibody. Mitochondrial fraction was confirmed with anti-porin antibody. **(C)** BY4741 transformants expressing VopE either constitutive (pAMU10, low copy vector, top panel) or inducible (pESCLEU, high copy vector, bottom panel) were subjected to spotting on selective solid media containing glucose or 2% glycerol. For liquid growth induction, liquid cultures were pretreated with galactose for 6 h before spotting.

To examine mitochondrial localization of VopE in yeast model, a yeast reporter strain BY4741-COX4-GFP was first constructed by transforming pCOX4-GFP in *S. cerevisiae* BY4741. COX4-GFP fusion protein (targeted to mitochondrial matrix) has been used to visualize mitochondrial morphology in yeast (Otsuga et al., 1998). Next, pVopE-mCherry was transformed into BY4741- COX4-GFP to generate BY4741-COX4-GFP-VopE-mCherry. In addition, VopE variants fused with mCherry (Table 1) were also transformed into BY4741-COX4-GFP. Upon induction, VopE-mCherry conferred growth inhibition in BY4741-COX4-GFP-VopE-mCherry (Figure S1), thus indicating that the fusion of mCherry at the C-terminus of VopE did not alter its activity. To visualize localization of mCherry tagged variants, the recombinant strains were harvested at 6 h following the addition of galactose and visualized by confocal microscopy. Similar to previous reports using mammalian cell lines, in yeast system also VopE and VopE^R125A^ localize to mitochondria whereas VopE^ΔMTS^ was detected in the cytosol (Figure 4A). Localization of VopE in mitochondria was further confirmed by sub cellular fractionation where presence of VopE-3XFLAG signal in yeast mitochondrial fraction was detected by immunoblot with anti-FLAG antibody (Figure 4B).

Certain *P. syringae* pv. *tomato* DC3000 effectors (HopAA1-1, HopAM1, HopAD1 etc.) when expressed in yeast have been shown to impair respiration and their toxicity enhanced on ethanol glycerol (EG) selective media (Munkvold et al., 2008). As VopE targets to mitochondria, it was therefore conceivable that VopE may alter respiration in yeast and show enhance killing of yeast cells on glycerol. But spotting of recombinant yeast strain harboring VopE cloned under constitutive or inducible promoters did not show any enhanced killing of yeast cells on selective media containing glycerol (2%; Figure 4C), thus indicating VopE does not impair yeast respiration.

### VopE maintains lethality in yeast mitochondrial GTPase deletion strains

**Figure 5:**
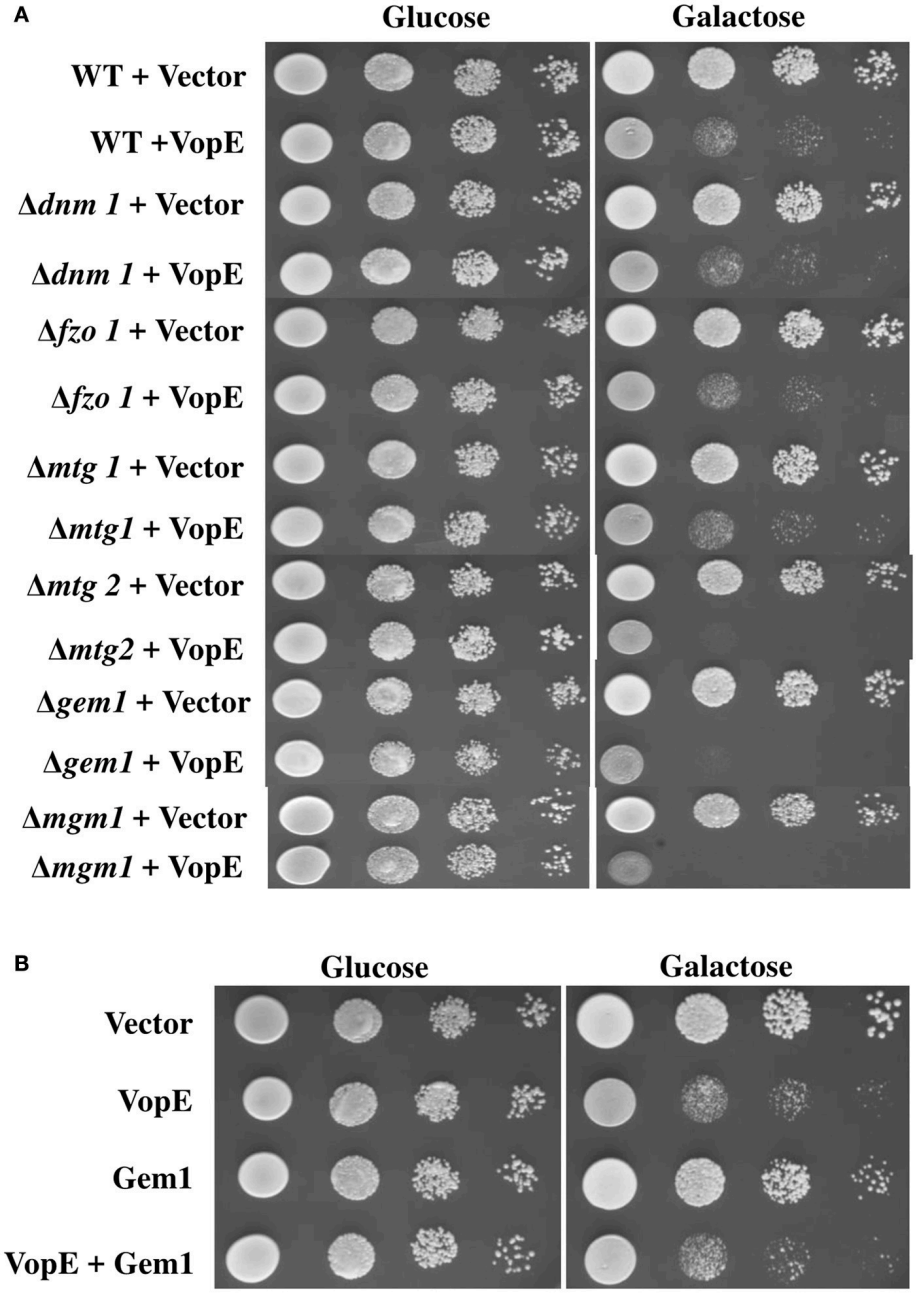
**Deletion or Over expression of Gem1 does not rescue yeast growth inhibition**. 10-fold serial dilutions of **(A)** indicated BY4741 deletion strains containing eitheir pESCLEU or VopE **(B)** BY4741 transformants carrying either Gem1 alone or Gem1 and VopE, were subjected to spotting on selective solid media with glucose or galactose.

Inhibition of mitochondrial clustering by VopE is mediated through the inactivation of Miro GTPases (Suzuki et al., 2014). The mammalian miro Rho GTPase ortholog in the budding yeast is Gem1p (Frederick et al., 2004). Like other miro proteins, Gem1p also contains two GTPase domains, a pair of calcium binding EF-hand motifs and C-terminal transmembrane (TM) domain. Both GTPase domains and EF-hand motifs are essential for Gem1p to maintain mitochondrial dynamics (Frederick et al., 2004). To examine whether the VopE dependent lethality in yeast system was also mediated by the Gem1p (yeast Miro GTPase), VopE was transformed into the yeast strain lacking Gem1p, BY4741Δ*gem*1 (Table 1) and growth was assayed after VopE expression. In addition, Gem1 was also over expressed in wild type yeast harboring VopE. Unexpectedly, VopE expression caused lethality in the gem1 deleted yeast strain and gem1 over expressed strain (Figures 5A,B). Conversely, Gem1, the yeast Miro GTPase is not targeted by VopE.

A battery of cellular components including several GTPases namely mitochondrial Rho GTPases (Miro I and Miro II), Fzo/mitofunsins (Mfn1 and Mfn2), Dnm1/Drp1/Dlp1, Mgm1/Opa1/Msp1 contributing to mitochondrial dynamics have been identified in yeast, flies, worms, and mammals (Shaw and Nunnari, 2002; Frederick and Shaw, 2007). VopE expression in a number of mitochondrial GTPase yeast deletion strains (Δ*dnm1*, Δ*fzo1*, Δ*mgm1*, Δ*mtg1*, Δ*mtg2*) produced lethality similar to that produced by VopE expression in the wild type yeast strain (Figure 5A).

### VopE mediated yeast growth inhibition was exacerbated in the presence of stressors: the signaling pathway

Many translocated bacterial effector proteins do not exhibit lethality in yeast model system either due to non-conservation of targets between yeast and mammalian system or because target (s) is conserved but it is dispensable and not rate limiting for yeast growth under normal conditions (Slagowski et al., 2008; Salomon et al., 2011). In latter cases, certain compounds, or environmental conditions known as “stressors” have been shown to increase the sensitivity of yeast strains toward different effectors and aid in the identification of effector mediated growth-retardation traits (Siggers and Lesser, 2008). The commonly employed stressors are (i) caffeine, a purine analog exerts pleiotropic effects in yeast and activates the CWI MAP kinase pathway. The molecule is highly toxic to the mutants of CWI pathway and Tor pathway (Parsons et al., 2004; Kuranda et al., 2006); (ii) Sorbitol, an osmotic stressor activates HOG (high osmolarity glycerol) MAP kinase pathway and toxic to mutants of the HOG MAPK pathway (Gustin et al., 1998; Hohmann, 2002); (iii) NaCl, an osmotic and ionic stressor that exhibits high level toxicity to the mutants of HOG MAPK pathway and ion homeostasis (Gustin et al., 1998; Hohmann, 2002; Auesukaree et al., 2009); (iv) tunicamycin, an inhibitor of protein glycosylation shows extreme toxicity to the mutants of unfolded protein response (UPR) pathway and mutants with cell wall defects (Parsons et al., 2004); (v) DTT, a reducing agent, is also known to induce ER stress (Papa et al., 2003; Bosis et al., 2011); (vi) 37°C heat stress, causes pleiotropic effects in yeast (Auesukaree et al., 2009). It has also been demonstrated that stressors also suppress the toxic activity of certain effectors. For example, lethality of *Coxiella burnetii* effector CBU0388-CetCb2 has been shown to be repressed by caffeine (Lifshitz et al., 2014).

**Figure 6:**
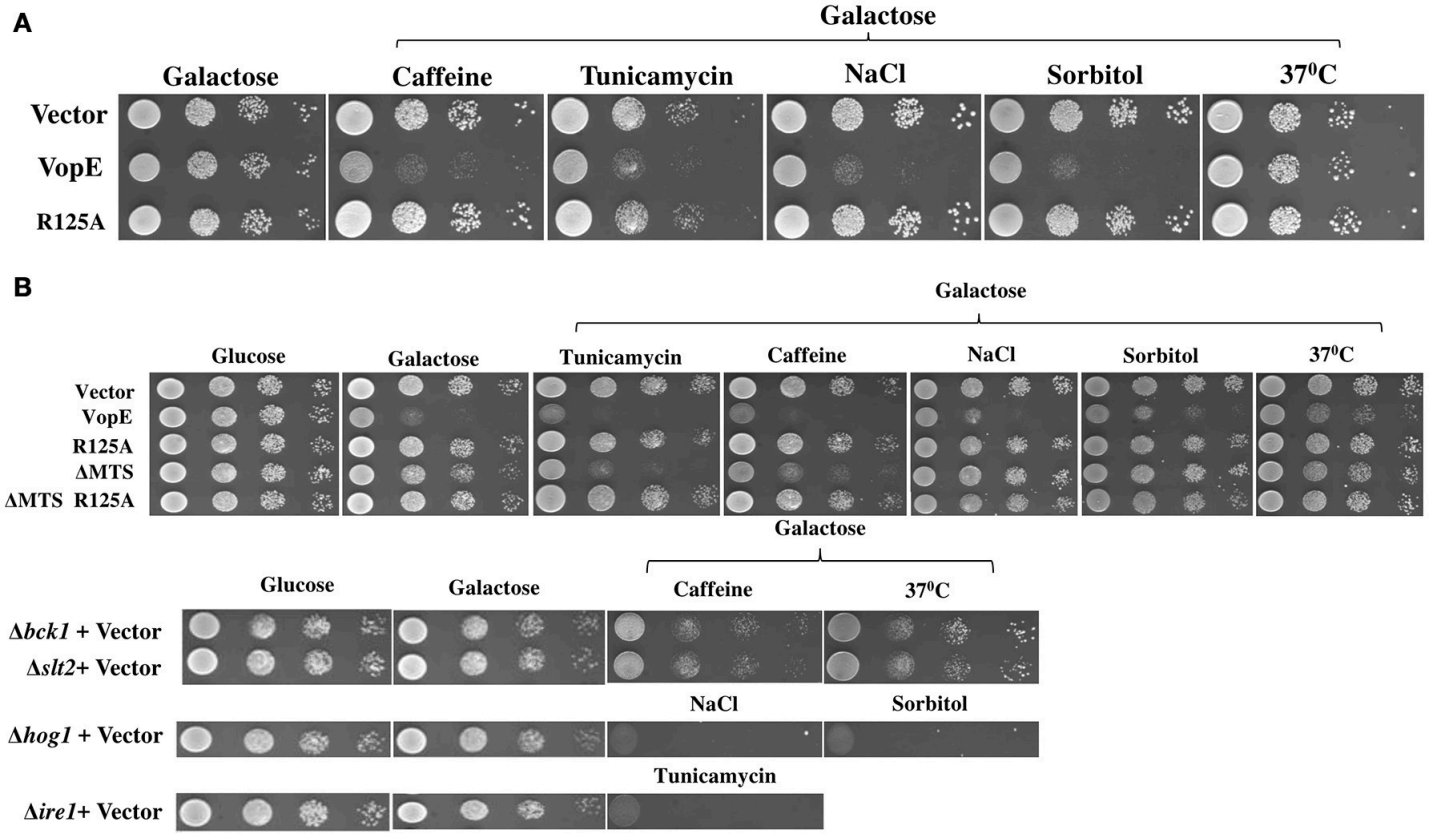
**Enhanced yeast toxicity under various stress conditions caused by VopE and VopE^**ΔMTS**^**. Yeast growth inhibition phenotypes of **(A)** W303-1A transformants carrying either VopE or VopE^R125A^ **(B)** BY4741 transformants carrying VopE or its derivatives were examined by spotting on solid selection media containing galactose (inducing) plate without or with stressors. pESCLEU used as cloning vector. Stressors used in this experiment were as follows: Caffeine (3 mM), Tunicamycin (0.3 μg/ml), NaCl (0.5 M), Sorbitol (1 M), 37°C. Corresponding stressor sensitized BY4741 deletion strains (Δ*bck1*, Δ*slt2*, Δ*hog1*, Δ*ire1*) were also spotted. Each experiment was repeated three times with similar results.

Since VopE showed no toxicity in *S.cerevisiae* strain W303-1A as compared to BY4741 in spotting assay (Figures 1A,B), the effect of various stressors on both *S. cerevisiae* strains W303-1A and BY4741 harboring VopE was next examined. For this purpose, the yeast strains were grown in the presence of some established stressors like caffeine (3 mM), NaCl (0.5 M), sorbitol (1 M), tunicamycin (0.3 μg/ml), and 37°C. Interestingly, VopE mediated lethality was observed in W303-1A in the presence of the stressors (caffeine, NaCl, sorbitol, and tunicamycin; Figure 6A). To further examine the cellular targets of VopE lacking MTS variant (VopE^ΔMTS^), BY4741-VopE^ΔMTS^ recombinant strain was grown in the presence of various stressors. We observed an increase in lethality of VopE^ΔMTS^ only in the presence of caffeine and tunicamycin (Figure 6B). Since yeast growth in the presence of caffeine and tunicamycin mediated stress condition require functional CWI and UPR signaling pathways respectively (Parsons et al., 2004; Kuranda et al., 2006), these results suggested that VopE and its mutant form (VopE^ΔMTS^) strongly perturbed the activation of CWI and UPR pathways. VopE also caused yeast growth failure in osmotic conditions reflecting attenuation of high osmolarity glycerol MAPK pathway. It may be noted that YopE, the *Yersinia* homolog of VopE, also targets CWI pathway (Kramer et al., 2007).

**Figure 7:**
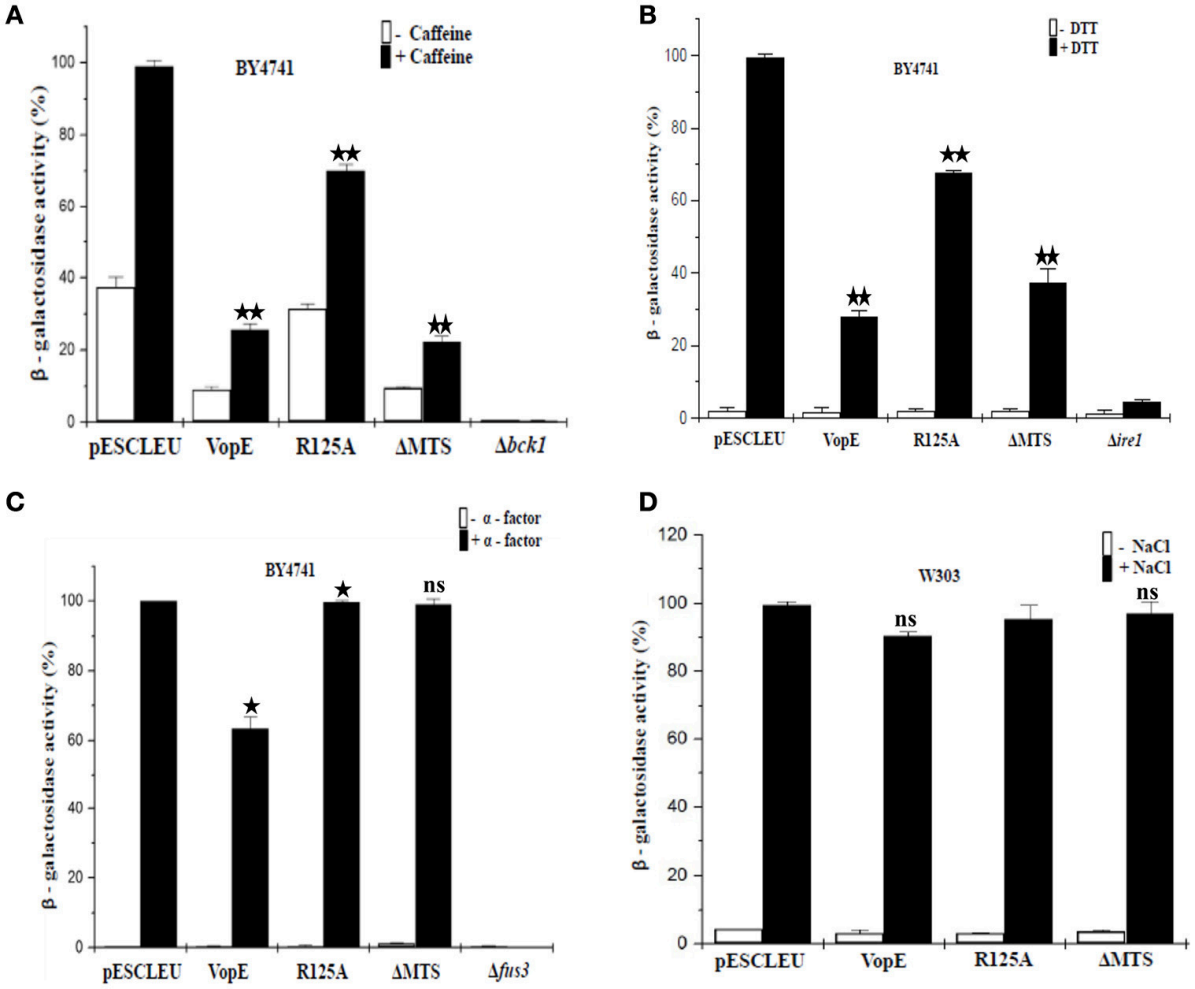
**Effect of VopE and VopE^**ΔMTS**^ on yeast MAPK pathways and ER stress response**. Under inducing conditions, **(A)** BY4741 yeast strains harboring 2X-Rlm1 regulated *lacZ* reporter plasmid and VopE or its derivatives were treated with 3 mM caffeine for 4 h. VopE and VopE^ΔMTS^ significantly affect the activation of CWI-MAPK pathway. **(B)** Unfolded protein response was examined by incubating BY4741 transformants carrying UPRE regulated *lacZ* reporter plasmid and VopE or its derivatives in presence of 2 mM DTT for 4 h. VopE or VopE^ΔMTS^ greatly hampered activation of UPR response. **(C)** Activation of P_FUS1_-regulated *lacZ* reporter was also monitored for BY4741 transformants carrying reporter plasmid and VopE or its derivatives in response to 5 μM α-factor for 2 h. Reduced activation of pheromone MAPK pathway has been observed in the presence of VopE. **(D)** For HOG-MAPK pathway, W303-1A strains containing 8 × *CRE*-regulated *lacZ* reporter and VopE or its derivatives were incubated in the presence of NaCl (0.5 M) for 1 h. Activation of reporter activity was not effected. Error bar depict the standard deviation from mean of three independent clones. All experiments were repeated three times with similar results. For VopE and VopE^ΔMTS^, an asterisk(s) indicates a statistically significant difference in mean values as compared to pESCLEU. For VopE^R125A^, an asterisk(s) indicates a statistically significant difference in mean values as compared to VopE (^*^p < 0.05; ^**^p < 0.001; ns-not significant; unpaired, two tailed student's *t*-test).

There are six MAPK cascades have been characterized in yeast mediating response to different stimuli namely (i) pheromone responsive pathway; (ii) filamentous growth pathway; (iii) high-osmolarity glycerol pathway; (iv) STE vegetative pathway; (v) nutrient starvation pathway; (vi) CWI pathway (Chen and Thorner, 2007; Arias et al., 2011). In order to identify the specific MAP kinase pathways which are majorly attenuated by VopE and its truncated form (VopE^ΔMTS^), we performed *lacZ*-reporter assay with recombinant yeast strains harboring VopE and VopE^ΔMTS^ along with *lacZ*-reporter plasmids of various MAPK signaling pathways (CWI, HOG, Pheromone) and ER stress pathways (UPR). The results clearly showed that VopE inhibited the activation of (i) pheromone signaling pathway (32% inhibition); (ii) CWI pathway (73% inhibition); (iii) unfolded protein response pathway (UPR; 70% inhibition). Interestingly, VopE^ΔMTS^ also significantly suppressed the activation of UPR and CWI pathways (Figures 7A–C).

Targeting multiple MAPK pathways has been reported for other effector proteins e.g., YopJ, HopX1 (Yoon et al., 2003; Salomon et al., 2012). It may be noted that no inhibition of *lacZ* reporter activity corresponding to HOG pathway was observed (Figure 7D), but toxicity of VopE was observed in the presence of NaCl and sorbitol (Figure 6A). Such discrepancy has also been reported earlier, caffeine increases the toxicity of XopE2, a T3SS effector of *Xanthomonas campestris*, but no inhibition of *lacZ* reporter activity corresponding to CWI pathway in the presence of caffeine was observed (Bosis et al., 2011).

### VopE^ΔMTS^ showed enhanced toxicity in yeast deletion mutants of CWI pathway

**Figure 8:**
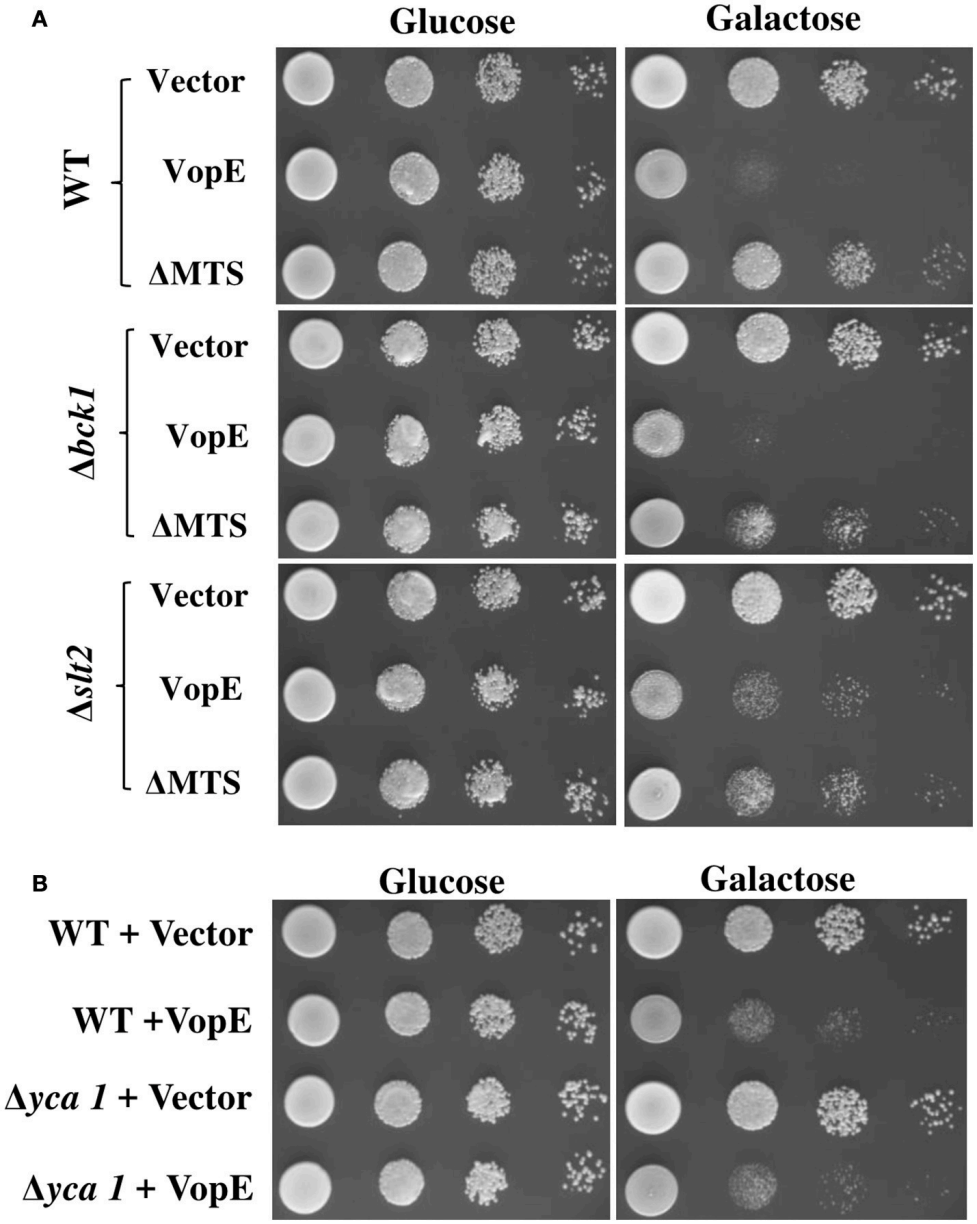
**Examination of VopE effect on CWI defective and metacaspase mutants. (A)** BY4741Δ*bck1* and BY4741Δ*slt2* yeast deletion mutant strains showed increased yeast growth inhibition phenotype toward VopE^ΔMTS^. **(B)** Over expression of VopE maintains toxicity in BY4741Δ*yca1* strain. All transformants were examined under solid selection media containing glucose or galactose. pESCLEU used as cloning vector.

To test whether yeast MAPK pathway components modulate the toxicity, we transformed VopE and VopE^ΔMTS^ in yeast strains deleted for MAPK components of different signaling pathways namely CWI (Δ*slt2*), High osmolarity glycerol (Δ*hog1*), Pheromone (Δ*kss1*), and sporulation (Δ*smk1*), and examined their growth under galactose inducing conditions. Of these, *slt2* deletion strain expressing VopE^ΔMTS^ showed increased yeast growth inhibition than wild type (Figure 8A, Figure S2).

The CWI pathway maintains cell wall integrity and gets activated in the presence of diverse arrays of external stimuli such as heat stress, mating pheromones, oxidative stress, pH stress, cell wall mutations, cell wall stressors, and endoplasmic reticulum stress all of which are known to cause cell wall stress (Levin, 2005; Arias et al., 2011). We further examined the effect of VopE and its truncated version on various yeast deletion mutants of CWI pathway. Accordingly, VopE and VopE^ΔMTS^ were transformed into yeast deletion mutants (Δ*bck1*, Δ*mkk1*, Δ*mkk2*, Δ*rlm1*, Δ*kdx1*) and the recombinant yeast strains were examined by spotting on solid agar plate. Unlike VopE, Δ*bck1* and Δ*slt2* mutants expressing VopE^ΔMTS^ exhibit increased yeast growth inhibition as compared to wild type strain (Figure 8A, Figure S2). An increased toxicity in absence of *bck1* and *slt2* further indicates that VopE and VopE^ΔMTS^ may activate a cellular response opposing the function of Bck1p and Slt2p. Such cases of hypersensitivity have been recorded with other effectors (e.g., OspF, XopE2, CBU1676, and CBU0885) as well (Kramer et al., 2007; Bosis et al., 2011; Lifshitz et al., 2014). It should be noted that lethality of VopE and VopE^ΔMTS^ was observed to be enhanced by tunicamycin, an UPR pathway activator. If VopE targets UPR pathway, then it should remain non-lethal in yeast deletion mutant of UPR pathway (Δ*ire1*). Instead, we observed maintenance of toxicity which further emphasizes that VopE does not target UPR pathway (Figure S3). It has been pointed out by Sessa and colleagues that tunicamycin is highly toxic to mutants with cell wall defects (Salomon et al., 2012). It is therefore possible toxicity of effectors targeting CWI such as VopE is exacerbated in the presence of tunicamycin.

Next, we wanted to investigate whether VopE mediated growth inhibition was due to initiation of apoptosis. Thus, we transformed VopE in yeast mutant Δ*yca1*, carrying a deletion in the metacaspase gene, *YCA1* and subsequently examined the viability of recombinant Δ*yca1* strain by solid agar spotting assay. If VopE targets YCA1, Δ*yca1* strain should remain viable in the presence of VopE. But we observed VopE maintained its lethality in *YCA1* deleted strain which is comparable to wild type strain background (Figure 8B). These observations further reinforce that VopE and VopE^ΔMTS^ interact with the components of cell wall maintenance related pathways.

### Co-expression of VopE^ΔMTS^ partially ameliorates the lethal effect of VopX in yeast

**Figure 9:**
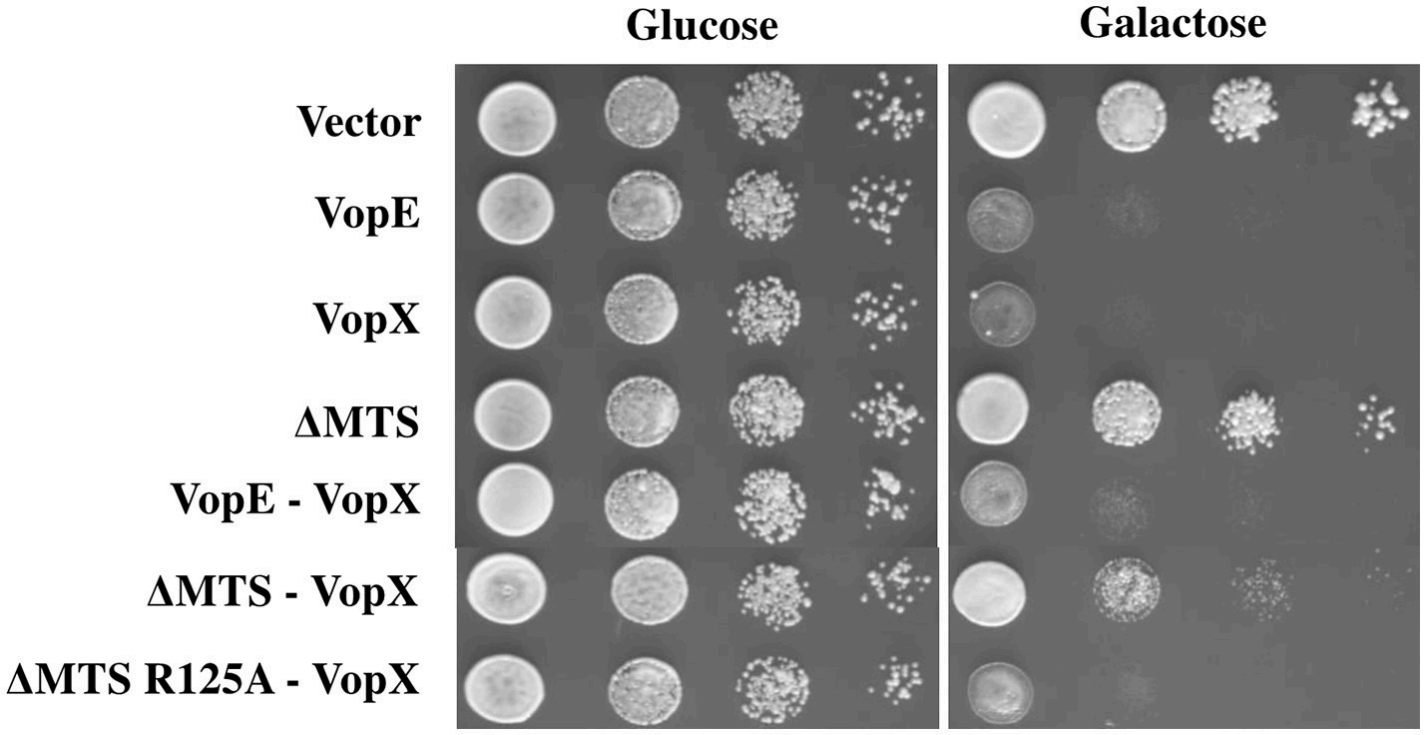
**Expression of VopE^**ΔMTS**^ suppresses yeast growth inhibition of VopX. (A)** BY4741 transformants carrying VopX and VopE^ΔMTS^, VopX and VopE^ΔMTS R125A^, or VopX and VopE were spotted on glucose and galactose plates. pESCLEU used as cloning vector for dual expression, where VopX and VopE variants cloned under *GAL10* and *GAL1* promoters, respectively.

Co-expression of two effectors in yeast model has proven valuable in identification of suppressor function of certain effectors over other. Previously, it has been shown that *L. pneumophila* effectors, SidD, and SidJ were able to counteract the activity of SidM and SdeA respectively (Tan and Luo, 2011; Havey and Roy, 2015). We investigated the outcome of co-expression of VopE^ΔMTS^ with another *V. cholerae* effector VopX targeting similar cellular pathway, the CWI pathway in yeast (Alam et al., 2011; Seward et al., 2015). The dual expression constructs (Table 1) were transformed to *S. cerevisiae* strain BY4741. The recombinant yeast strains were subjected to solid agar spotting under galactose inducing condition. We observed a partial suppression of VopX mediated toxicity in dual expression condition by VopE^ΔMTS^ only (Figure 9). To further confirm the specificity, we co-expressed the functionally inert variant of VopE^ΔMTS^ harboring the substitution of catalytic arginine at position 125 with alanine (VopE^ΔMTS R125A^) along with VopX. We observed that VopE^ΔMTS R125A^ did not inhibit VopX mediated toxicity further supporting the hypothesis that VopE^ΔMTS^ mediates suppression of VopX toxicity (Figure 9).

To summarize, the present work unfolds many additional features on functional aspects of VopE by exploiting yeast model system. These are as follows: (i) identification of several critical residues in GAP domain and N-terminal region of VopE conferring lethality in yeast system; (ii) VopE variant lacking mitochondrial localization signal possesses partial lethality; (iii) screening of yeast deletion library against VopE and its derivative VopE^ΔMTS^ results in the identification of hypersensitive strains; (iv) VopE and VopE^ΔMTS^ modulates CWI-MAPK pathway in yeast; (v) co-expression of VopE^ΔMTS^ and VopX causes functional perturbation of the latter.

## Discussion

In this study the budding yeast has been exploited as a model organism for functional exploration of VopE, a T3SS effector protein of *V. cholerae*. Previously, yeast has been used as a model organism to study the activity of several other *V. cholerae* T3SS effector proteins (Tripathi et al., 2010, 2013; Bankapalli et al., 2015; Seward et al., 2015).

It is now evident that one important host organelle targeted by several pathogens is the mitochondria (Jiang et al., 2012). Usually, proteins targeted to the mitochondria harbor specific signal sequence known as mitochondrial targeting signal (MTS) at the N-termini contributing to their import into mitochondria. Site-directed mutagenesis of MTS reveals the identification of critical residue (s) involved in the mitochondrial transportation of certain effectors (e.g., Map, EspF) (Nagai et al., 2005; Papatheodorou et al., 2006). In case of some proteins, PorB (*Neisseria gonorrhoeae*), and VacA (*Helicobacter pylori*), removal of N and C-termini did not affect their mitochondrial localization, indicating that signals are not restricted at the termini only (Kozjak-Pavlovic et al., 2009; Foo et al., 2010; Jiang et al., 2012). Like many other mitochondria targeted effectors, localization of VopE is also dependent on the predicted mitochondrial-targeting sequence spanning 23 amino acids at the N-terminus. Deletion of MTS composed of 23 amino acids (VopE^ΔMTS^) or substitution of leucine with glutamate at position 4 in the MTS (VopE^L4E^) abolishes the localization of VopE in mitochondria. Unlike many mitochondria targeted effectors, VopE does not promote the death of host cells. Functionally, it modulates innate immune signaling through mitochondrial dynamics (Suzuki et al., 2014).

Effectors having localization signals of various cellular compartments (e.g., Map, ExoS, YopE etc.) still maintain toxicity even after removal of the signal (Zhang and Barbieri, 2005; Papatheodorou et al., 2006; Isaksson et al., 2009; Geissler et al., 2010). Interestingly, one mitochondrial targeted effector of enteropathogenic *E. coli* known as Map is found to be more toxic after removal of MTS in yeast model system (Papatheodorou et al., 2006). Keeping this in mind, we wanted to investigate and garner additional insight on the functionality of VopE by exploiting yeast model system. Our data clearly indicated that ectopic expression of VopE and VopE^ΔMTS^ caused toxicity to yeast cells.

VopE is a member of bacterial GAP family protein. Structural and functional analysis of certain bacterial GAPs clearly establishes a communication network between several conserved residues imparting the GAP function. By employing *in silico* analysis followed by site-directed mutagenesis and functional evaluation in yeast model system, we have identified several residues other than R125 contributing to the lethal function of VopE. It should be noted that natural sensitivity of BY4741 to VopE mediated toxicity further aid in the identification of critical residues in VopE.

There is now a large body of evidence on strategies used by pathogens to control host signaling pathways especially kinase signaling pathways (Krachler et al., 2011). Various kinase signaling pathways such as nuclear factor-kB (NF-kB), mitogen-activated protein kinase (MAPK), phosphatidylinositol 3-kinase (PI3K), and p21-activated kinase (PAK) are hijacked by different phylogenitically related and unrelated pathogens (Krachler et al., 2011). Interestingly, T3SS effectors of some of these pathogens (e.g., YopJ, VopA, OspF) have been shown to inhibit MAPK kinase signaling pathways in yeast model system as well (Yoon et al., 2003; Trosky et al., 2004; Kramer et al., 2007). With the help of stressors, β-galactosidase assay and yeast deletion strains, we have revealed that VopE and its variant VopE^ΔMTS^ modulate CWI-MAPK pathway in yeast model system, but the mechanism is not clear. Yeast two-hybrid analysis of VopE^ΔMTS^ against prenylation defective GTPases (Rho1, Rho1^Q68H^, Rho2, Rho2^Q65H^, CDC42, CDC42^Q61L^) failed to detect any possible interaction (data not shown).

Being the principal signaling pathway maintaining the architecture of yeast cell wall, the structural and functional aspects of CWI MAPK pathway have been studied in great detail (Levin, 2005). There is now a growing list of diverse effector proteins (e.g., OspF of *Shigella*; Icm/Dot effectors of *C. burnetti*; VopX of *V. cholerae*; YopE of *Y*. *pestis*) perturbing the function of CWI-MAPK pathway in yeast model system (Kramer et al., 2007; Lifshitz et al., 2014; Seward et al., 2015). As evidenced, T3SS effectors of different bacteria target and alter the function of various components of CWI pathway. While OspF inhibits the phosphorylation of Slt2, the terminal MAP Kinase of CWI pathway (Kramer et al., 2007), VopX modulates Rlm1 function and Rlm1 mediated downstream genes expression by interacting components at the top of MAPK cascade (Alam et al., 2011; Seward et al., 2015). Interestingly, CBU0388, CBU0885, and CBU1676, effectors of *C. burnetti* interact differentially with the various MAP Kinases of CWI pathway. While yeast deletion mutants (Δ*bck1* and Δ*mpk1*) of CWI pathway exhibit hypersensitivity upon expression of CBU1676 and CBU0885, the same mutants result complete suppression of CBU0388 lethality (Lifshitz et al., 2014). Our observation of VopE mediated functional modulation of CWI-MAPK pathway will extend the growing list of bacterial effectors targeting this important cellular pathway in yeast. Finally, our co-expression of two effectors interfering CWI-MAPK, VopE^ΔMTS^ and VopX, exhibited the suppression of lethality of the latter in the yeast model.

As demonstrated previously, VopE targets Miro GTPase and causes functional modulation of the innate immune system (Suzuki et al., 2014). The Miro ortholog in the budding yeast is Gem1p (Frederick et al., 2004). If VopE lethality in yeast is linked to Gem1p, then VopE should not exert toxicity either in BY4741Δ*gem1* or its toxicity should be mitigated upon over expression of gene encoding Gem1p. Our data clearly demonstrated that VopE maintained its lethal property in BY4741Δ*gem1* as well as BY4741 harboring Gem1p over-expression construct, thereby indicating lethality of VopE is not mediated by Gem1p. Interestingly, substitution of critical arginine finger residue at position 125 (R125) by alanine (R125A) conferred non-functionality in VopE and VopE^ΔMTS^ further corroborated that GAP function is required for the lethality of these proteins in yeast system. Currently, the targeted GTPase(s) of VopE and its variant in yeast model system is not known. This warrants further investigation.

## Author contributions

SR conceived the idea. SR and LB designed the experiments. LB carried out all the experiments. RM repeated the experiments. SR wrote the manuscript. All the authors gave their editorial input and approved the final manuscript.

## Funding

This work was supported by the CSIR program on supra institutional project (SIP-BSC0210E). LB and RM received research fellowships from Department of Biotechnology (DBT, India) and CSIR project (UNSEEN-CSIR) respectively. Funder has no role in the design of study, analysis and interpretation of data and in writing the manuscript.

### Conflict of interest statement

The authors declare that the research was conducted in the absence of any commercial or financial relationships that could be construed as a potential conflict of interest.
