# Supplementary figures and images for: VopE, a Vibrio cholerae Type III Effector, Attenuates the Activation of CWI-MAPK Pathway in Yeast Model System
Source: Front Cell Infect Microbiol. 2017 Mar 20;7:82. doi: 10.3389/fcimb.2017.00082 (PMC5357651; doi:10.3389/fcimb.2017.00082)

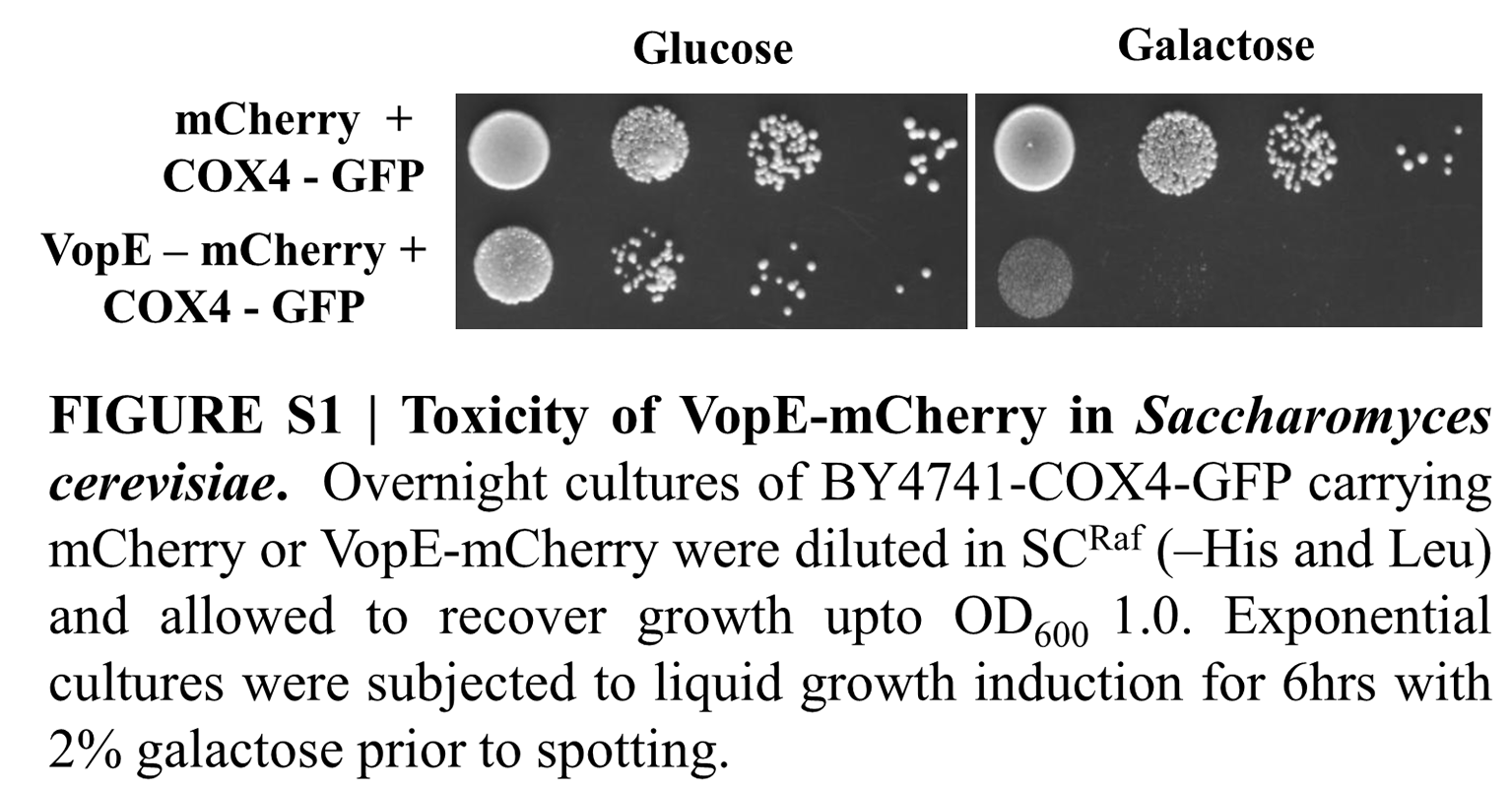

Supplement: Supplementary file 1 [file Image1.TIF]

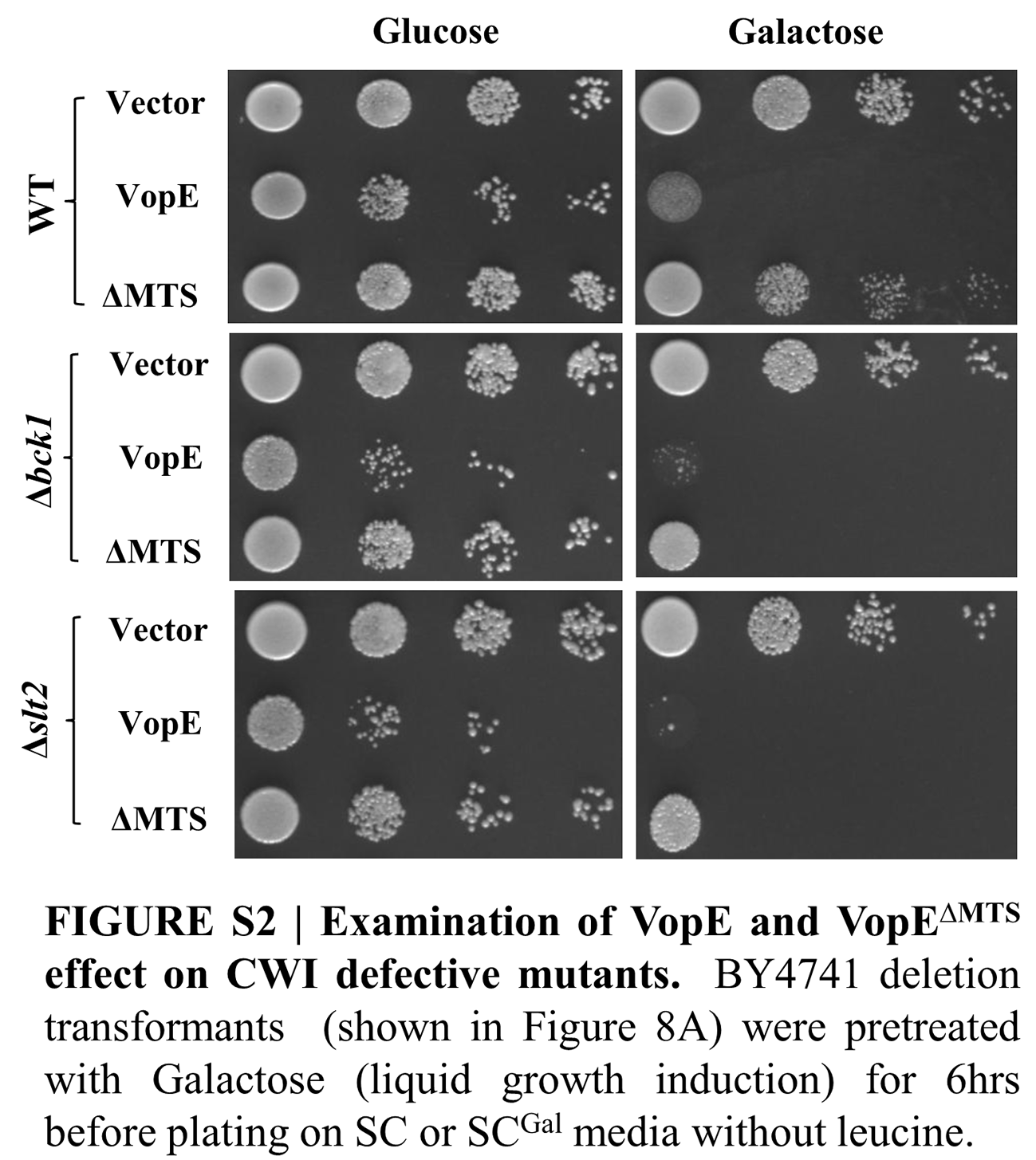

Supplement: Supplementary file 2 [file Image2.TIF]

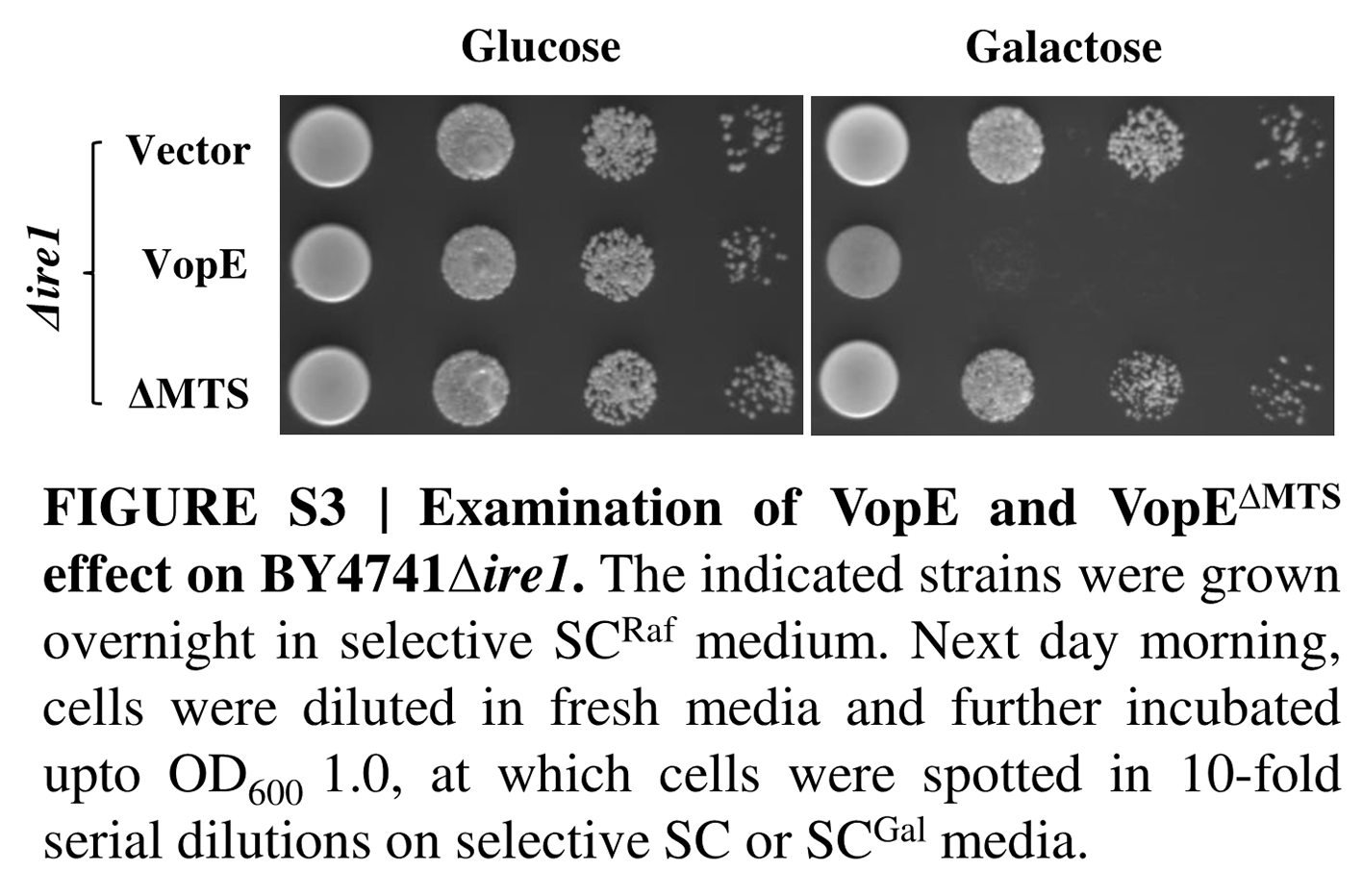

Supplement: Supplementary file 3 [file Image3.TIF]
